# Effects of Metformin on Symptoms of Polycystic Ovarian Syndrome Among Women of Reproductive Age

**DOI:** 10.7759/cureus.3203

**Published:** 2018-08-25

**Authors:** Moiz Artani, Mohammad Faisal Iftikhar, Shehroz Khan

**Affiliations:** 1 Department of Community Health Sciences, Jinnah Medical and Dental College, Karachi, PAK

**Keywords:** metformin, pakistan, polycystic ovary syndrome, women

## Abstract

Background

Metformin is a highly preferred drug that is used to treat the symptoms of polycystic ovarian syndrome among women. Pakistan is facing a continuous rise in the prevalence of polycystic ovarian syndrome. Therefore, the determination of the effectiveness of the drug in this condition is considered ideal as per the presenting situation.

Objective

The aim of the study was to determine the effects of metformin in patients with polycystic ovarian syndrome.

Methods

This cross-sectional study has investigated the influence of metformin on the ability to conceive, body weight, mood swings, energy levels, menstrual irregularities, and acne and hirsutism. As per the inclusion criteria, 100 women were evaluated from the outpatient clinics of Jinnah Postgraduate Medical Centre and Civil Centre, Karachi, from November 2016 to January 2017. A structured, pre-tested questionnaire was used for data collection.

Results

The study group had a mean age of 27.2 ± 4.75 years. Outcomes reported by study participants suggested a significant influence of metformin on menstrual irregularities (p 0.046), acne and hirsutism (p<0.001), mood swings (p<0.001), and daily energy levels (p<0.001). Findings further proposed that metformin does not produce a significant impact on the ability to conceive (p 0.096) and in the change in body weight (p 0.073) of the patients.

Conclusion

Metformin has been realized to have a significant role in dealing with the symptoms of polycystic ovarian syndrome. It is recommended to conduct more in-depth and longitudinal research on the long-term effects of the drug and compliance among these women.

## Introduction

Polycystic ovarian syndrome or PCOS is recognized as one of the most prevalent endocrine conditions that affect a considerable proportion of women of reproductive age [1]. It is primarily indicated as the morphological alteration of ovaries in the form of multiple cysts [2-6]. The dysfunction further involves the biochemical and metabolic factors of hyperandrogenism and fertility issues [6]. It is characterized as a heterogeneous abnormality due to the variation in its clinical presentation and underlying complications [7].

Studies have determined that the prevalence of PCOS ranges between 2.2%-26.7% [3-4]. According to studies conducted by Gul et al. [8] and Nazir et al. [9], the prevalence of polycystic ovarian syndrome is continually rising in Pakistan. One study has also estimated, in its findings, that the prevalence of PCOS tends to be higher by 40.9% among the Pakistani women with fertility issues [10]. Despite the increasing prevalence, the etiology of PCOS has not been documented in a clear manner [7]. The indistinctness is more attributed to the variation in the clinical presentation of the condition, which may include weight gain and irregularities in the menstrual cycle. Moreover, this syndrome has been identified as a preceding factor to chronic conditions like diabetes mellitus [1,8], which continues to rise in parallel with PCOS. It is observed that the disease commonly occurs among young females, affecting their reproductive abilities [11].

Diabetes mellitus is a common condition in South Asia. The association between diabetes mellitus and PCOS has contributed to the concern for female health in Pakistan. Studies have clearly indicated that PCOS, along with glucose intolerance, marks serious effects on reproductive health, yet it has also been deemed that the impact is not limited to the gynecological issues only. Rather, PCOS has also been found to disturb the metabolism of the affected individual, moreover revealing that insulin resistance may have a potential role in inducing the pathogenesis of polycystic ovarian syndrome [12].

With respect to the indications, Metformin has been recognized as the most preferred treatment modality for PCOS. It is known to decrease the circulating levels of androgens and body weight, which eventually manages the symptoms and cysts [13-14]. Due to the popularity of metformin for treating PCOS [15-17], this study was aimed to determine the effects of metformin in patients with polycystic ovarian syndrome. It has particularly assessed the six variables of PCOS, including the ability to conceive, body weight, mood swings, energy levels, menstrual irregularities, and acne and hirsutism. The study has determined if metformin is a significant treatment for helping with these symptoms in PCOS. It has been primarily hypothesized that metformin may significantly enhance the ability to conceive, changes in body weight, and reduction in menstrual irregularities among the affected women of Karachi.

## Materials and methods

Study design and population

This is a cross-sectional study conducted at the outpatient clinics of the department of obstetrics and gynecology at Jinnah Postgraduate Medical Centre and Civil Centre, Karachi, from November 2016 to January 2017. Women at or above 18 years of age, with an upper limit of those who have not undergone menopause, were included in the study. It was particularly ascertained from patient records that each participant has had a clinical diagnosis of PCOS from the hospital and has been using metformin as per a doctor’s prescription. The duration of metformin use differed for each woman but was accounted for in the results. Women with any other co-morbidity such as diabetes, asthma, hypertension, history of stroke, or on any drug other than metformin were excluded from the study.

Data collection

A questionnaire was adapted from the World Health Organization Quality of Life Instruments (WHOQOL-BREF), where questions were modified and added as per the study requirement [18]. WHOQOL-BREF is a questionnaire for determining health-related quality of life and is validated in Pakistan. Our questionnaire was also pre-tested on 20% of the study population. The survey collected data, including demographics, menstrual history, quality of life, and family history from the respondents. A separate section was added to the questionnaire for women who have been trying to conceive during their treatment with metformin, solely to assess any improvement in ovulation and ability to conceive. Another section documented the pre and post-therapy symptoms with respect to the use of the drug.

The effects of metformin on symptoms before and after use were determined from the same participant. Recall bias is a possibility that is inherent in the design of cross-sectional studies. As our study population was regularly followed up by physicians for changes in symptoms, we were able to assist them to recall their initial symptoms and compare them to the results of metformin therapy. We also took the assistance of medical records and previous prescriptions to ensure a minimal recall bias. Furthermore, we asked trained female physicians to interview all the participants to avoid embarrassment and to aid in creating a comfortable environment where women can answer questions without hesitation or shame.

This survey evaluated the local populace of Karachi, yet it was not translated into the local language. Rather, each contributor was interviewed by one of the investigators for clarity. Each participant was informed about the aim and outcomes of the study and was selected on the basis of a confirmed diagnosis of polycystic ovarian syndrome by their doctors.

Ethical consideration

Written informed consent was obtained from the respondents. Ethical approval for the research was provided by the review board of Jinnah Medical and Dental College. The collected information was anonymized, coded, and analyzed on the Statistical Package for the Social Science (SPSS) version 19 (IBM Corp., Armonk, NY, US).

Statistical analysis

The SPSS software was used for analysis. Means and median were calculated for normally distributed and non-normally distributed quantitative variables, respectively. For categorical variables, frequency and percentage were reported. For evaluating the measures of association, the chi-square test was used. The Fisher Exact test was used where the cell count was <5 for a variable. A p-value of 0.05 was considered significant.

## Results

The study evaluated 100 women with age ranging between 18 and 48 years. The mean age of the study participants was 27.2 ± 4.75 years. All the participants were residents of Karachi. The ratio of married to unmarried patients was 1.7:1. Table 1 presents the general attributes of the study group.

**Table 1:** Descriptive statistics for the study population (N=100)

Parameter	Variable	Frequency
Marital Status	Married	63
	Unmarried	37
Age of Menarche	< 12 years	18
	12 – 13 years	49
	14 – 15 years	22
	>15 years	11
Family History for PCOS	Positive	43
	Negative	57
Duration of Metformin Use	1 – 4 months	49
	5 – 12 months	18
	1 – 2 years	13
	More than 2 years	20

Table 2 shows that prior to metformin therapy, 34% of the study population had problems in conceiving while the number decreased to 24% after the use of metformin (p-value=0.09). As for the primary indication of PCOS, menstrual irregularities were observed by 100% of the study group. Though, after the use of metformin, 69% of the study respondents indicated relief from the complaints of menstrual cycle irregularity. Of these, 81% of the women reported weight gain with PCOS while 19% reported weight loss. With the administration of metformin, the numbers turned to 61% for weight gain and 39% for weight loss among the respondents. With the highest recorded proportion in the study, with the exception of menstrual irregularities, mood swings were indicated by 92% of the participants. However, after treatment with metformin, 64% reported the problem. Similarly, 83% of the participants reported acne and hirsutism, which decreased to 58% following the administration of metformin. Moreover, 54% of the women reported low energy and fatigue before the treatment whereas the number decreased to 37% after Metformin. It was observed that prior to metformin therapy, 34% of the study population had problems in conceiving while the number decreased to 24% after the use of metformin though the association was not statistically significant (p 0.096).

**Table 2:** Comparative observations before and after the administration of metformin

Parameters	Variable	Before Metformin (%)	After Metformin (%)	Significance (p=0.05)
Ability to Conceive	Normal	22	32	0.096
	Problems	34	24	
	Not Recorded	44	44	
Changes in Weight	Gain	81	61	0.736
	Lost	19	39	
Mood Swings, Lethargy & Depression	Yes	92	64	<0.001
Daily Energy	High	46	63	<0.001
	Low	54	37	
Menstrual Irregularities	Yes	100	31	0.046
	No	0	69	
Acne & Hirsutism	Increase	83	58	<0.001
	Reduction	0	25	
	None	17	17	

The outcomes of the study indicates that metformin does not mark a significant influence on the ability to conceive (p 0.096) and change in body weight (p 0.736) of the patients, however, it suggested a significant impact of the drug administration on relieving acne and hirsutism (p<0.001), indications of mood swings (p<0.001), daily energy levels (p<0.001), and menstrual irregularities (p 0.046) as presented in Table 2.

## Discussion

In this study, the use of Metformin has presented its association with relief in acne and hirsutism, an improvement in the occurrence of mood swings, a reduction/resolution of menstrual irregularities, and an increase/decrease in daily energy levels. The drug was not associated with the ability to conceive and reduction of body weight. As per the literature, the effectiveness of metformin has emerged as a subject of great interest in the 21st century. Hashim et al. [19] reviewed the CENTRAL and PubMed database for evaluating the reproductive benefits of this drug. The assessment of presenting randomized controlled trials (RCTs) and reports of meta-analysis indicated that the empirical evidence is not sufficient enough to indicate any association between the ability to conceive and the administration of metformin among patients of PCOS [19-20].

It has been determined in this study that the reproductive abilities of a woman with PCOS may not gain any benefits from metformin treatment. However, other studies have indicated that a combination therapy of metformin and gonadotropins may enhance ovulation induction among PCOS patients [21]. The mechanism of action of these two administrations is unknown and, thus, requires more research on the subject. Similarly, as seen in the results of this study, metformin does not appear to have a marked impact on the body weight of patients. Yet, it has been asserted in several studies that it may enhance weight loss and decrease weight gain in PCOS when given in combination with a supporting drug [22]. Despite two insignificant findings, the study has presented a significant improvement in the problems and irregularities of the menstrual cycle among the patients suffering from the polycystic ovarian syndrome. These results are supported by other findings, as metformin has been realized to have relieving effects on menstrual irregularities, which suggests its role in enhancing endocrine production and secretions [23]. It should be noted that as per the primary hypothesis, the study has recommended with statistical significance that metformin has been effective in treating indications of menstrual irregularities.

Among all the indications of polycystic ovarian syndrome, acne and hirsutism are considered the most embarrassing factors of the disease that have been noted to deteriorate the emotional well-being and self-esteem of the affected women [24]. This study has indicated a beneficial and significant impact of metformin on these two conditions. Recent studies have also supported these outcomes by indicating the consistent efficacy of this drug treatment [25-26]. Contemporary research has extensively supported the role of metformin in enhancing physical health. Additionally, the effectiveness of the drug on mental health has also been under scrutiny for quite a few years. This study has recognized a positive impact of Metformin on the mood instability and energy levels of PCOS patients.

This study has presented limitations in terms of small sample size, study centers, and study period, yet the findings have presented an insight into the effectiveness of the drug. Information on the dosage of the drug was taken from the participants. Since this information was self-reported and we could not validate whether the patients were prescribed the same dosage, we removed the dosage variable from the analysis. It is recommended that an evaluation shall be conducted on a larger scale that may also record the impression of dosages and the effects of long-term metformin therapy on PCOS.

## Conclusions

Metformin has been realized to have a significant role in relieving the symptoms of polycystic ovarian syndrome. Recent researches and the outcomes of this study have suggested that a more comprehensive investigation must be performed to determine the role of metformin as a monotherapy and in combination with other supporting drugs.
